# Comparison of the Anterior Limit of the Dentition in Patients Treated with Self-Ligating Straight-Wire, Conventional Straight-Wire and Standard Edgewise Appliances

**DOI:** 10.5402/2012/748758

**Published:** 2012-07-01

**Authors:** Luca Lombardo, Paolo Ficara, Ivano Maltoni, Lorenz Moser, Maria Paola Guarneri, Giuseppe Siciliani

**Affiliations:** Postgraduate School of Orthodontics of Ferrara, Ferrara University, via Montebello, 31 Ferrara 44100, Italy

## Abstract

The aim of this study was to identify and compare any differences in the position of the anterior limit of the dentition provoked by three different types of orthodontic mechanics: traditional edgewise, straightwire and self-ligating. A sample of 54 patients (selected from a group of 289 patients) possessed a range of Little's Irregularity Index values for the upper and lower dental arches between a minimum of 6.5 and a maximum of 13.5 at T0, and corresponding final values no greater than 2 and hence of minimal irregularity. The 54 patients were subdivided into three groups according to the type of brackets used in their treatment: Group 1 was composed of 24 patients treated using the self-ligating technique, Group 2 of 15 patients treated using a conventional straight-wire technique (Roth) and Group 3 of 15 patients treated using the standard edgewise technique. Cephalometric tracings were performed on laterolateral teleradiography. Group 1 value was found to be nonsignificant, whereas significant labial inclination was noted in Groups 2 and 3 (*P* < 0.05). A significant labial inclination of the upper incisors was also evidenced in all three sample groups.

## 1. Introduction

Besides reduction of dental malocclusion, the fundamental aims of orthodontic treatment are to achieve a stable dynamic functional equilibrium not subject to relapse, and to guard against future damage to the temporomandibular joint [1]. The introduction of straight-wire appliances in the 1970s was an evolutionary leap in this sense; they were designed to achieve three-dimensional control of dental position without the need for bends in the wire. However, none of these affirmations have ever been confirmed in the literature and, furthermore, over recent decades many torque prescriptions have been proposed for the same teeth, depending on the technique in question.

Moreover, relatively few studies into the labiolingual inclination of the incisors, except for those by Pandis [2] and Scott et al. [3], have analysed the inclination of the incisors in people undergoing orthodontic treatment. Indeed, various factors, such as symphyseal morphology, intra- and extraoral neuromuscular structures, condyle guides, and the occlusal plane, which are closely linked to the anterior limit of the dentition, need to be taken into account for correct positioning of the incisors to be achieved [4–7]. In particular, control of the anterior area is determined by an equilibrium between the external (upper and lower orbicular muscles) and internal musculature (i.e., the tongue) [8–10].

During the course of the last decade, the orthodontic world has seen another evolution in the development and rapid diffusion of self-ligating systems. The fundamental advantages of this type of appliance are the elimination of conventional means of ligation (elastic or metallic ligatures) and a considerable reduction in the friction generated between archwire and bracket [11]. Moreover, authors [12] have noted that lowering the level of friction consents the use of light forces, allowing the system to work together with the orofacial musculature [13], bringing about a more physiological repositioning of the teeth, in harmony with the biological structures such as muscles, tongue, bone, and soft tissue [9, 15]. However, wide-ranging and robust scientific evidence to back up these claims is still conspicuous by its absence. 

*The aim of our study* was, therefore, to identify any differences in the maintenance of the position of the anterior limit of the dentition between three different orthodontic mechanics: conventional edgewise, straightwire, and self-ligating.

## 2. Materials and Methods

The following records of 289 patients random selected, taken from the databases of two operators, were analysed:

- objective assessment,
- analysis of models (at T0 and T1),
- photographic records,
- radiographic analysis (OPT and laterolateral teleradiography),
- cephalometric analysis.

Patients featuring the following criteria were excluded: mixed dentition, extraction cases, impacted teeth, agenesis, open bite, deep bite, orthodontic surgery cases, labial incompetence, use of miniscrews, and class II or class III skeletal malocclusion.

Thus, 54 class I malocclusion patients were selected and divided into the following three groups according to the treatment mechanics and appliances used during the treatment.

 Group 1 (Dsl): 24 patients treated using the Damon self-ligating technique.
 Group 2 (SW): 15 patients treated using a conventional straight-wire appliance (Roth).
 Group 3 (Tw): 15 patients treated using the standard Tweed-Merrifield edgewise technique.

Homogeneity of the three groups was ascertained by assessing their malocclusion characteristics and determining the severity of the same, the latter by means of Little's Irregularity Index [16]. The Little's Index was calculated for the upper and lower jaws using an apposite software developed by Mutinelli et al. [17], and previously documented in the literature.

Upper and lower Little's Indexes were measured, and cephalometric tracings were performed for the three patient groups at:

 T0: prior to orthodontic treatment (bonding),
 T1: after treatment (debonding).

Models analysis and upper and lower Little's Index evaluation were performed at T0 and T1 by means of the abovementioned software. Homogeneity of the subjects considered was ensured by selecting patients within a range of Little's Index values from 6.5 (minimum) to 13.5 (minimum) at T0. In our opinion, rather than using a statistical mean of each group, a method which would not exclude the presence of subjects with very high or low Little's Index values within the group, this approach permitted real homogeneity of malocclusion severity between the groups to be achieved. 

In order to compare the efficacy of treatment, the same criterion used for sample selection at T0 was adopted at T1. In fact, none of the 54 subjects selected had final upper or lower Little's Index values greater than 2, and, therefore, the sample possessed minimal irregularity [16].

No intra- or extraoral intermaxillary appliances, that is, elastics, lip bumpers, expanders, or traction devices and no stripping were employed during the course of the orthodontic treatment. The self-ligating appliance fitted in Group 1 patients featured a 0.22-inch slot with a standard prescription value of torque equal to −1° on the lower incisors and +12° on the uppers. The Roth straight-wire appliance used in Group 2 patients likewise featured a 0.22-inch slot, but was devoid of first, second, and third degree information. The last archwire used in all three Groups was a stainless steel wire of lesser dimensions than the slot, that is, a 0.19∗0.25 SS.

Cephalometric measurements were taken from tracings on teleradiograms in laterolateral projection at T0 and T1. All measurements and tracings were performed by the same operator using Dolphin software. Three values were employed.

IMPA: the angle formed between the long axis of the lower incisor and the mandibular plane passing through points Go and Gn.

UIA: the angle formed between the long axis of the upper incisor and the bispinal plane passing through points SpA and SpB.

Finally, in order to consider any variations in occlusal plane inclination (OPI), the angle formed between the occlusal plane and the bispinal plane was evaluated.

## 3. Statistical Analysis 

To characterise the sample from a descriptive perspective, means and standard deviations were calculated. Wilcoxon's nonparametric test was used to evaluate any differences between pre- and posttreatment values of IMPA, UIA, and OPI. In order to compare the variance in these values in the three groups, ANOVA (analysis of variance), was used after the delta (difference between pre- and posttreatment) value was calculated for each subject. Finally, the three groups were compared at T0 and T1, also by means of ANOVA. All statistical tests were performed using JMP software, Version 7.0.1, SAS Institute Inc., Cary, NC, 1989–2007.

## 4. Results 

The following Tables (Tables 1 and 2) report the descriptive statistical analysis of the three groups, as well as the variation in relative IMPA and UIA values between T0 and T1 in the three groups (*P* < 0.01). 

Group 1 possessed initial and final IMPA values of 88.7° (SD 6.31°) and 92.05° (SD 6.36°), respectively. 

In Group 2, mean pre- and posttreatment IMPA values were, respectively, 89.08° (SD 7.82) and 95.97° (SD 7.12°), a statistically significant difference (*P* < 0.05). Corresponding Group 2 values for UIA were 108.4° (SD 8.49) and 114.6° (SD 3.92°), also a statistically significant difference (*P* < 0.05).

Finally, IMPA values for Group 3 were 87.2° (SD 6.25°) prior to treatment and 98.3° (SD 8.1°) afterwards, a statistically significant difference (*P* < 0.01). Initial and final UIA values were 103.2° (SD 7.99°) and 111.05° (SD 6.2°), respectively, another statistically significant difference (*P* < 0.01). 

*Analysis of variance* was then performed by means of ANOVA, pairing the pre- and posttreatment IMPA values for all three Groups.  Table 3 shows a comparison of the three Groups, evaluating the differences in IMPA and UIA between T0 and T1. The difference between the three groups as regards the lower incisor inclination at T0 was not statistically significant (*P*  value > 0.7235). The difference between the three groups as regards the upper incisor inclination at T0 was not statistically significant (*P*  value > 0.1098).

Subsequently, we compared the three Groups at T1, again using ANOVA. The final IMPA values of 92.05° in Group 1, 95.97° in Group 2, and 98.3° in Group 3 yielded a statistically significant difference (*P* < 0.05). Finally, Table 4 shows the inclination of the occlusal plane (OP) with respect to the bispinal plane for the three Groups at T0 and at T1 (Table 4). Regarding the inclination of the occlusal plane (OP), the mean pretreatment and posttreatment values were 9.31° (SD 2.88°) and 9.71° (SD 3.40°), respectively, in Group 1, the difference between the two being nonsignificant. Analysis of variance, by means of ANOVA, was then used to compare data regarding the variation in the inclination of the occlusal plane (OPA) relative to the bispinal plane using delta (Δ), that is, the difference between pre- and posttreatment values.  Table 3 shows a comparison of the three Groups as regards the difference in pre- and posttreatment OPA. The value for Group 1 was 0.40°, for Group 2 it was 2.25°, and for Group 3 it was 0.42°; the difference between the Groups was not found to be significant.

Finally, the inclination of the occlusal plane of the three groups were compared at T0 and at T1, that is, before and after treatment, again using ANOVA. The difference between the groups as regards OP inclination at T0 was not found to be significant (*P*  value > 0.2006). Likewise, no statistically significant difference was found between OP inclination at T1 (*P*  value > 0.2009).

## 5. Discussion

In this work, we used dental arch crowding as a tool for evaluating the efficiency of three different bracket types and biomechanical systems in managing the position of the upper and lower incisors. Little's Irregularity Index [16] was employed to select the degree of crowding that subjects required to be placed in the three test groups, thereby guaranteeing homogeneity of the sample as regards the severity of malocclusion. These values were set at a minimum of 6.5 mm and a maximum of 13.5 mm, while the reduction of these values seen at T1, values recorded at around 1 mm, verified the efficacy of all three treatment strategies. In fact, the results of this study highlight that, between T0 and T1, the three biomechanical systems produced respective lower incisor labial inclination of 3.35° in Group 1 (edgewise), 6.88° in Group 2 (conventional straightwire), and 11.06° in Group 3 (self-ligating straightwire). This labial inclination was found to be nonsignificant in Group 1, but significant in Groups 2 and 3 (*P* < 0.05). A similar pattern was seen as regards labial inclination of the upper incisors: 4.29° in Group 1, 6.27° in Group 2, and 7.85° in Group 3, except that in this case the difference between T1 and T0 was found to be significant in all three sample groups studied. These results suggest that all three types of appliance permit correction of crowding by a similar mechanism: labial inclination of the incisors and modification of the arch form.

In the recent and not-so-recent literature, many studies have confirmed a statistically significant proinclination of the incisors during treatment in samples treated using edgewise, straightwire and self-ligating techniques. In the study by Pandis et al., in 2007 [2], Damon 2 brackets were compared with conventional edgewise brackets, both of which were found to produce a significant degree of lower incisor proinclination during treatment (Damon = from 93.70° to 101.11°, Edgewise = from 95.66° to 101.88°). However, no statistically significant differences between the two systems were found. Similarly, in the study by Scott et al. [3], published in 2008, labial inclination of the lower incisors was noted to occur during both self-ligating and conventional treatments, the difference between pre- and posttreatment values being 2.34° for the edgewise treatment and 1.73° for the Damon approach; once again the difference between the two groups was not found to be significant.

In the 2006 study by Pandis et al. [19] on the expression of upper incisor torque, a significant difference between sample and control groups was reported, but no significant difference was noted between groups treated with Roth-type and conventional appliances. These authors highlighted the role of the “play” between the narrow archwire and the slot in determining a loss of torque, as well as emphasising the influence of other factors, in common with other authors such as Van Loenen et al. and Vigorito et al. [18, 19], who cited, in particular, tooth morphology, lip posture, and a difference in angulation between the crown and root axes of the incisors. In another study, Germane et al. [20] concluded that dental morphology varies progressively from the anterior to the posterior sectors in both arches, and that the surface of each tooth varies considerably between the occlusal and gingival margins. 

Our study, on the other hand, did reveal statistically significant differences between treatment groups at T1, implying a better capacity of self-ligating mechanics to control the position of the lower incisors. Various factors could be behind this behaviour, including modification of the arch form and lack of homogeneity between the groups due to the different torque prescriptions employed. Indeed, although the incisor torque was the same in Groups 1 and 2, it differed in the edgewise brackets, being zero in this case. Furthermore, variations in tip and torque were present in the lateroposterior sectors. Moreover, as the final archwire used in all three groups measured 0.19∗0.25 SS and the bracket slot 0.22, a loss of torque expression of around 10° [20–22] due to the interaction between the two was inevitable. As stated by Ugur and Yukay [21], the loss of a certain quantity of torque between edgewise appliances and pretorqued brackets means that individual patient variation needs to be considered when planning treatment. This is why, even in pretorqued appliances, the necessity of archwire bending to adjust the final torque may not be eliminated entirely.

The theoretical principles of straightwire and self-ligating mechanics, particularly the latter, are based on the sliding of the archwire within the bracket slots and on exploitation of a very light system of forces to move the teeth using highly technological wires and friction-lowering techniques, thereby altering the relationship of forces between muscular structures such as the tongue, lips, and facial muscles. This change determines a new equilibrium of forces that would seem to cause arch form remodelling and dental repositioning guided by biological, rather than heavy orthodontic, forces [23]. The use of a light system of forces and sliding mechanics would also appear to consent better control of vertical forces and permit good control of the occlusal plane during treatment. 

In order to test this hypothesis, another objective of our study was to measure any variation in occlusal plane inclination (OPA) with respect to the bispinal plane between T0 and T1, which was found to be nonsignificant for any of the treatment techniques considered. Furthermore, no statistically significant difference was evidenced between the treatment types, although it should be mentioned that Group 2 underwent a greater variation in occlusal plane inclination with respect to Groups 1 and 3, whose OP excursion was minimal. 

## 6. Conclusions

The results of this study suggest the following conclusions.

Based on their Little's Index scores, all three appliances tested were found to be clinically efficacious in resolving crowding between T0 and T1.

All three appliances tested caused labial inclination of the lower incisors during the course of treatment. This labial inclination was not found to be significant in Group 1, but was in Groups 2 and 3. No significant differences were noted concerning the upper incisors. 

No statistically significant differences were found in any group as regards inclination of the occlusal plane between T0 and T1.

## Figures and Tables

**Table 1:** Variation in IMPA angle in Groups 1, 2, and 3.

	Group 1	SD	Group 2	SD	Group 3	SD
IMPA at T0	88,7	±6.3	89	±7.8	87	±6.25
IMPA at T1	92,1	±6.3	95	±7.1	98	±8.13
*P* value^∗^	NS		<.05		<.05	

**Table 2:** Variation in upper incisor inclination (UIA) with respect to bispinal plane in Groups 1, 2, and 3.

	Group 1	SD	Group 2	SD	Group 3	SD
UIA at T0	109	±9.7	108	±8.4	103	±7.9
UIA at T1	113	±6.4	114	±3.9	111	±6.1
*P* value^∗^	<.05		<.05		<.05	

**Table 3:** Comparison of the three groups by evaluation of the mean differences in IMPA and UIA values at T0 and T1.

	IMPA t0	IMPA t1	Δ	IS t0	IS t1	Δ	PO t0	PO t1	Δ
Group 1	88,7	92,05	3,35	109,1	113,4	4,29	9,31	9,7	0,40
Group 2	88,87	95,96	6,88	107,9	114,6	6,27	7,57	9,82	2,25
Group 3	87,22	98,29	11,06	103,2	111,05	7,85	7,37	7,37	0,42
*P* value^∗^	NS	<.05	<.01	NS	NS	NS	NS	NS	NS

**Table 4:** Description of angle between occlusal plane (OPA) and bispinal plane at T0 and T1 in Groups 1, 2, and 3.

	Group 1	SD	Group 2	SD	Group 3	SD
OPA at T0	9,3	±2.88	7,5	±3.3	7,36	±5.04
OPA at T1	9,7	±3.4	9,8	±3.49	7,78	±3.85
*P* value^∗^	NS		NS		NS	
